# NOP53 undergoes liquid-liquid phase separation and promotes tumor radio-resistance

**DOI:** 10.1038/s41420-022-01226-8

**Published:** 2022-10-31

**Authors:** Jie Shi, Si-Ying Chen, Xiao-Ting Shen, Xin-Ke Yin, Wan-Wen Zhao, Shao-Mei Bai, Wei-Xing Feng, Li-Li Feng, Caolitao Qin, Jian Zheng, Yun-Long Wang, Xin-Juan Fan

**Affiliations:** 1 Department of Radiation Oncology, The Sixth Affiliated Hospital, Sun Yat-sen University, Guangzhou, Guangdong 510655 P.R. China; 2 Guangdong Provincial Key Laboratory of Colorectal and Pelvic Floor Diseases, The Sixth Affiliated Hospital, Sun Yat-sen University, Guangzhou, Guangdong 510655 P.R. China; 3 Guangdong Institute of Gastroenterology, Guangzhou, Guangdong 510655 P.R. China; 4 Center for Reproductive Medicine and Department of Gynecology & Obstetrics, The First Affiliated Hospital, Sun Yat-sen University, Guangzhou, 510080 P.R. China; 5 Department of Pathology, The Sixth Affiliated Hospital, Sun Yat-sen University, Guangzhou, Guangdong 510655 P.R. China

**Keywords:** DNA damage response, Mechanisms of disease

## Abstract

Aberrant DNA damage response (DDR) axis remains the major molecular mechanism for tumor radio-resistance. We recently characterized liquid-liquid phase separation (LLPS) as an essential mechanism of DDR, and identified several key DDR factors as potential LLPS proteins, including nucleolar protein NOP53. In this study, we found that NOP53 formed highly concentrated droplets in vivo and in vitro, which had liquid-like properties including the fusion of adjacent condensates, rapid fluorescence recovery after photobleaching and the sensitivity to 1,6-hexanediol. Moreover, the intrinsically disordered region 1 (IDR1) is required for NOP53 phase separation. In addition, multivalent-arginine-rich linear motifs (M-R motifs), which are enriched in NOP53, were essential for its nucleolar localization, but were dispensable for the LLPS of NOP53. Functionally, NOP53 silencing diminished tumor cell growth, and significantly sensitized colorectal cancer (CRC) cells to radiotherapy. Mechanically, NOP53 negatively regulated p53 pathway in CRC cells treated with or without radiation. Importantly, data from clinical samples confirmed a correlation between NOP53 expression and tumor radio-resistance. Together, these results indicate an important role of NOP53 in radio-resistance, and provide a potential target for tumor radio-sensitization.

## Introduction

Radiotherapy (RT) plays a central role in curing multiple types of solid cancer [1, 2]. The therapeutic effects of RT are traditionally associated with the introduction of DNA double-stranded breaks (DSB), the most lethal form of DNA damage [3, 4]. Nevertheless, cells displaying intrinsic radio-resistance survive after RT, resulting in a poor clinical outcome [5, 6]. Various studies have revealed that these radio-resistant cancer cells displayed accelerating DNA damage response (DDR) signaling [4, 7]. In response to DNA damage, the tumor suppressor protein p53 is rapidly activated, which upregulates genes associated with cell cycle arrest or cell death [8]. Meanwhile, many DNA repair factors were recruited to the DSB to form a high concentrated repair center for DNA damage repair [9].

Liquid-liquid phase separation (LLPS) or condensation, is a physicochemical process by which macromolecules solution such as proteins or nucleic acids separate into a dense phase and a dilute phase [10, 11]. Recently, LLPS has been recognized as an essential molecular mechanism underlying the formation of membraneless organelles in cells, e.g., nucleoli and paraspeckle [12, 13]. The driving force of LLPS is the multivalent interactions among macromolecules, one of which is mediated by the intrinsically disordered regions (IDR) of proteins [14, 15]. LLPS plays a key role in a myriad of cell functions, including transcription, chromatin organization, autophagosome formation, etc. [16, 17]. Recently, we and other groups have reported that DNA repair proteins may undergo LLPS to facilitate DSB repair [13, 18, 19]. For example, Silvia M L Barabino, et al. reported that the multifunctional DNA/RNA-binding protein fused in sarcoma (FUS) underwent LLPS upon DNA damage and its LLPS was necessary for the initiation of DDR [20]. In addition, our recent study identified several DDR factors as potential LLPS proteins, including NOP53 [19].

NOP53 (also known as GLTSCR2 or PICT1) is one of the nucleolar proteins with crucial functions in cell growth and homeostasis, including ribosome biogenesis and DNA damage response [21, 22]. NOP53 knockdown decreased both the presence of γ-H2AX at the nuclear and the activation of multiple DDR factors, which further sensitized cells to DNA damage [23]. Previously, NOP53 seems to act as a tumor suppressor by stabilizing p53 in response to ribosomal stresses [24]. On the other hand, NOP53 was found promoting tumorigenesis by interfering RPL11/MDM2/p53 axis [25]. Nucleolus is a well-known membraneless organelles driven by LLPS. Several nucleolar proteins, such as Fibrillarin (FBL) and Nucleophosmin (NPM1), has been characterized to undergo LLPS, which contribute to the formation of nucleolar multilayered biomolecular condensate [26]. However, whether NOP53 forms liquid-like condensates and its roles in radio-resistance remain unclear.

Here, using in vivo and in vitro assays, we determined NOP53 as a LLPS protein in cellular nucleolus. Furthermore, IDR1 was found to be required for NOP53 condensates formation and multivalent-arginine-rich linear motifs (M-R motifs) were essential for the nucleolus localization of NOP53. Mechanically, NOP53 suppressed irradiation-induced p53 activation, thereby enhancing radio-resistance of colorectal cancer (CRC) cells. These results provide new insight into the role of NOP53 in DNA damage response and radio-resistance.

## Results

### NOP53 was localized in nucleoli and showed liquid-liquid phase separation property in cells

**Fig. 1:**
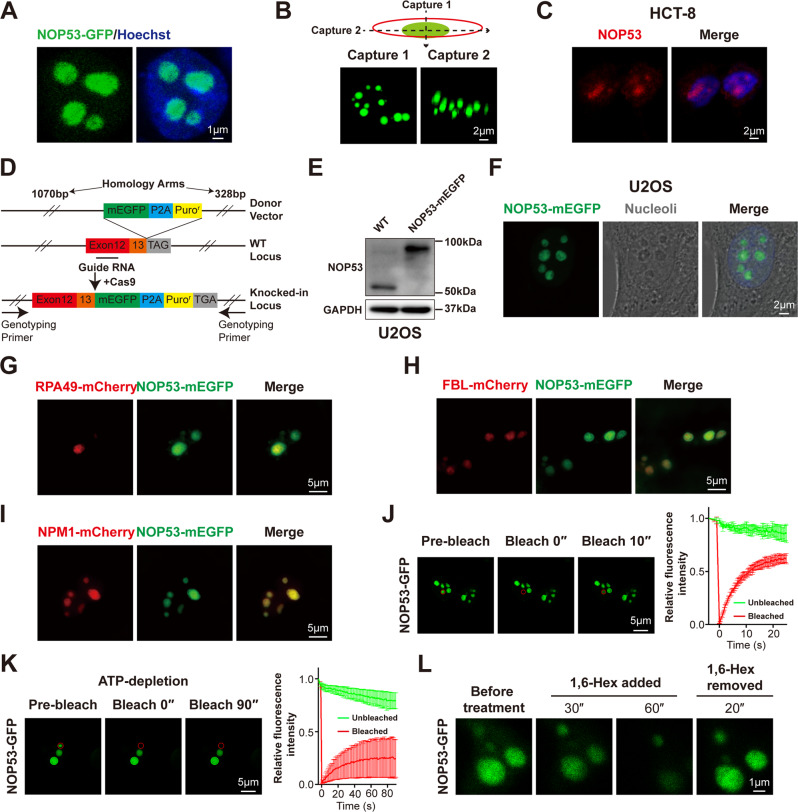
NOP53 was localized in nucleoli and showed liquid-liquid phase separation property in cells. **A** NOP53-GFP showed puncta in nucleus of HEK293T cells. **B** HEK293T cells were imaged using Z-stack modular of Zeiss LSM880 to show the 3-dimentional image of NOP53 puncta. **C** Immunofluorescence of endogenous NOP53 in HCT-8 cells. **D** Experimental schematic of mEGFP-KI U2OS cell line. **E** mEGFP-KI U2OS cell line was verified by western blotting. **F** Endogenous NOP53-mEGFP showed puncta in the nucleolus of mEGFP-KI U2OS cell line. **G**–**I** NOP53-mEGFP was colocalized with NPM1-mCherry, but not with RPA49-mCherry and FBL-mCherry. HEK293T cells were transfected with plasmids for 24 h before observation using Nikon-Structured IIIumination Microscopy (N-SIM). **J** FRAP of NOP53-GFP puncta in HEK293T cells. The bleached punctum was highlighted with a red circle. **K** FRAP of NOP53-GFP puncta in ATP-depleted cells. The bleached punctum was highlighted with a red circle. For (**J** and **K**), *n* = 3 punctum analyzed in 3 independent experiments. Data are mean ± SD. **L** NOP53-GFP droplets were disrupted by 10% 1,6-hexanediol and recovered after removal of 1,6-hexanediol.

Firstly, we investigated whether NOP53 formed puncta in living cells. NOP53-GFP was ectopically expressed in HEK293T cells and observed using a confocal microscope. In agreement with bioinformatic prediction, NOP53-GFP formed spherical condensed puncta in cells (Fig. 1A, B). Consistently, immunofluorescence assay using an antibody against NOP53 showed endogenous NOP53 formatting puncta in the nucleus of HCT-8, HeLa, and U2OS cells (Fig. 1C and Supplementary Fig. 1A, B). To further verify whether endogenous NOP53 forms puncta in live cells, we knocked an open reading frame of mEGFP into the C-terminal of NOP53 at its genomic locus of U2OS cells (Fig. 1D, E and Supplemental Material). Using live-cell fluorescence microscopy, we observed that endogenous NOP53-mEGFP formed puncta in nucleoli (Fig. 1F). To further visualize the subnucleolar localization of NOP53, we co-overexpressed NOP53 with nucleolar proteins including RNA polymerase I subunit 49 (RPA49), FBL or NPM1. These nucleolar proteins are component of the fibrillar center (FC), the dense fibrillar component (DFC) and the granular component (GC) of nucleolus, respectively [26]. We found that NOP53 was colocalized with NPM1, suggesting its subnucleolar localization at GC region (Fig. 1G–I). These results indicate that NOP53 forms puncta in nucleoli.

We next analyzed if NOP53 puncta showed LLPS property. Fluorescence recovery after photobleaching (FRAP) assay was used to investigate the dynamic exchange between puncta and diffused phase. We observed rapid fluorescence recovery of NOP53-GFP after photobleaching (Fig. 1J and Supplementary Fig. 1C). Meanwhile, depletion of adenosine triphosphate (ATP) resulted in a remarkable reduction of FRAP rate, demonstrating that the rapid molecular exchange of NOP53 puncta was an energy-dependent process (Fig. 1K). Importantly, when incubated with 1,6-hexanediol, NOP53 puncta rapidly dissolved but reformed shortly after the removal of 1,6-hexanediol (Fig. 1L). Collectively, these results indicate that NOP53 forms liquid-like condensates and localizes in nucleoli.

### Recombinant NOP53-mEGFP protein undergoes LLPS in vitro

**Fig. 2:**
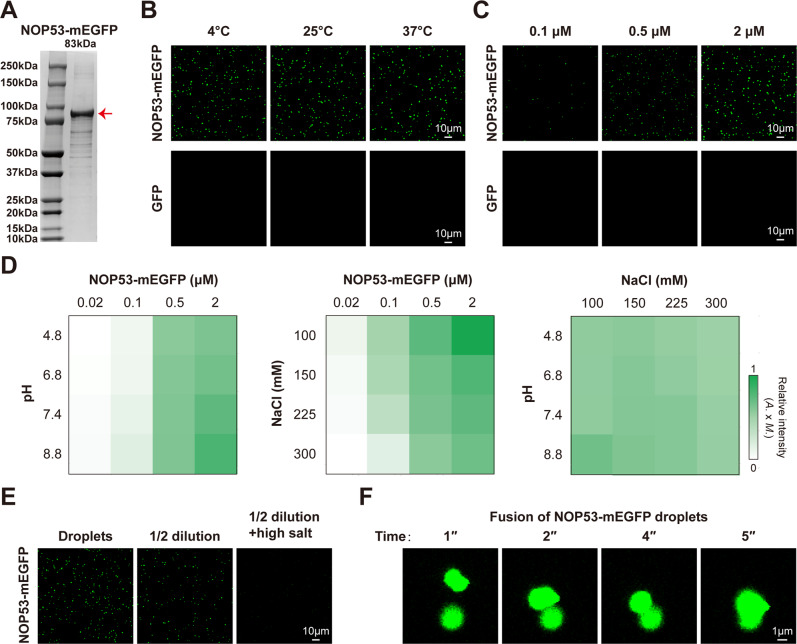
Recombinant NOP53-mEGFP undergoes LLPS in vitro. **A** Coomassie staining of purified NOP53-mEGFP protein. **B** NOP53-mEGFP protein formed droplets at different temperature. Two micromolar protein was used. **C** NOP53-mEGFP droplets that formed in buffers containing 150 mM NaCl and 25 mM Tris-HCl (pH 7.4) were observed with confocal microscopy. **D** The impact of protein concentration, NaCl concentration and pH on the formation of NOP53-mEGFP droplets. The fluorescence intensity of droplets is presented as the mean intensity × area. **E** NOP53-mEGFP droplets were disrupted by dilution and increasing NaCl concentrations. Droplets formed in buffer containing 2 μM NOP53-mEGFP and 150 mM NaCl at pH 7.4; high salt, 500 mM NaCl. **F** Two in vitro-formed NOP53-mEGFP droplets fused to form a larger droplet.

To further investigate whether NOP53 undergoes LLPS in vitro, we expressed and purified recombinant NOP53-mEGFP and GFP protein (Fig. 2A and Supplementary Fig. 2A). NOP53-mEGFP protein was diluted in a buffer containing 150 mM NaCl and 25 mM Tris-HCl (pH7.4) and incubated for 10 min at different temperature. Consistent with our hypothesis, NOP53 formed droplets at different temperature (Fig. 2B) in a concentration-dependent manner (Fig. 2C and Supplementary Fig. 2B). In addition, Na^+^ concentration and pH are also known to affect phase separation. We observed that LLPS of NOP53 was enhanced by high pH, but disrupted by high Na^+^ concentration (Fig. 2D). We next examined whether NOP53 phase separation was reversible. Lowering the protein concentration in the droplet-containing solution reduced NOP53 droplets, while increasing the concentration of NaCl further disrupted the droplets (Fig. 2E), suggesting that NOP53 LLPS was reversible upon changes of physiological conditions. Through time-lapse imaging, we observed different NOP53 droplets fusing to form a larger droplet, which was consistent with its liquid-like property (Fig. 2F). Taken together, these studies reveal that recombinant NOP53-mEGFP protein undergoes LLPS in vitro.

### The IDR1 drives the LLPS of NOP53

**Fig. 3:**
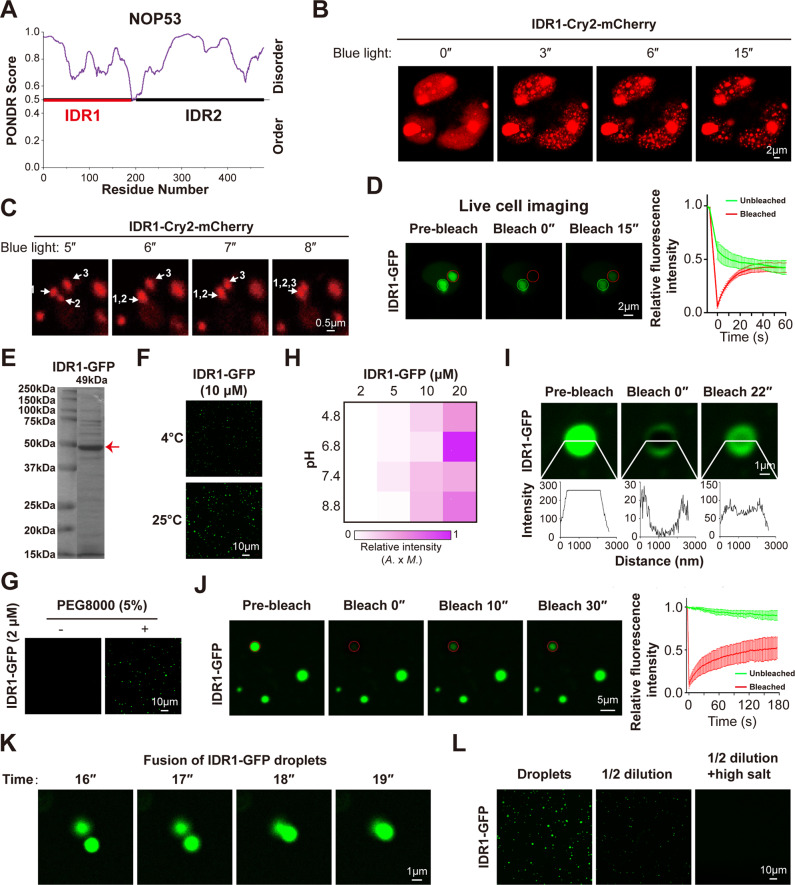
IDR1 drives the LLPS of NOP53. **A** The disordered region of NOP53 was analyzed with PONDR (www.pondr.com). **B** IDR1-Cry2-mCherry was expressed in cells, which were stimulated with blue light to induce condensation. **C** IDR1-Cry2-mCherry droplets fused to form a larger droplet upon stimulation with blue light. **D** FRAP of IDR1-GFP puncta in HEK293T cells. **E** Coomassie staining of purified NOP53-IDR1-GFP protein. **F** NOP53-IDR1-GFP protein formed droplets at different temperatures. Ten micromolar protein was used. **G** PEG-8000 enhanced the formation of NOP53-IDR1-GFP droplets. Two micromolar protein was used. **H** The impact of protein concentration and pH on the formation of NOP53-IDR1-GFP droplets. The fluorescence intensity of droplets is presented as the mean intensity × area. **I**, **J** FRAP of IDR1-GFP droplets in vitro. **K** Two in vitro-formed IDR1-GFP droplets fused to form a larger droplet. **L** IDR1-GFP droplets were disrupted by dilution and increasing NaCl concentrations. Droplets formed in buffer containing 10 μM IDR1-GFP and 150 mM NaCl at pH 7.4; high salt, 500 mM NaCl. For (**D** and **J**), *n* = 3 punctum analyzed in 3 independent experiments. Data are mean ± SD.

OptoIDR is an optogenetic tool containing Cry2 protein, a protein tends to aggregate under blue light stimulation. If IDR has a high LLPS capacity, OptoIDR would undergo rapid and strong LLPS after blue light treatment [19, 27]. Time-lapse imaging revealed that NOP53-Cry2-mCherry recombinant protein formed droplets upon blue light stimulation, and the fusion between different droplets was observed at the same time (Supplementary Fig. 3A, B). PONDR analysis showed that NOP53 held two IDRs (Fig. 3A). OptoIDR was used to further investigate whether both or any one of two IDRs in NOP53 mediates its phase separation. Interestingly, recombinant protein containing NOP53-IDR1 and Cry2-mCherry formed droplets rapidly after blue light stimulation, whereas that containing NOP53-IDR2 failed to form droplets (Fig. 3B and Supplementary Fig. 3C). Most importantly, blue light-induced NOP53-IDR1-Cry2-mCherry droplets were observed to fuse with each other (Fig. 3C). These results revealed that IDR1 is more important for NOP53 phase separation. Furthermore, ectopically expressed NOP53-IDR1-GFP was observed to form large puncta that were colocalized with nucleoli (Supplementary Fig. 3D) and contained high FRAP rate (Fig. 3D and Supplementary Fig. 3E). In addition, purified recombinant NOP53-IDR1-GFP protein underwent LLPS as efficient as the full-length NOP53 protein in vitro (Fig. 3E–H and Supplementary Fig. 3F). Consistently, droplets of recombinant NOP53-IDR1-GFP had the expected liquid-like properties including rapid FRAP, the fusion capacity and the reversibility of condensates formation (Fig. 3I–L). Overall, these results suggest that IDR1 drives the LLPS of NOP53.

### NOP53 undergoes LLPS independent of the nucleolus

**Fig. 4:**
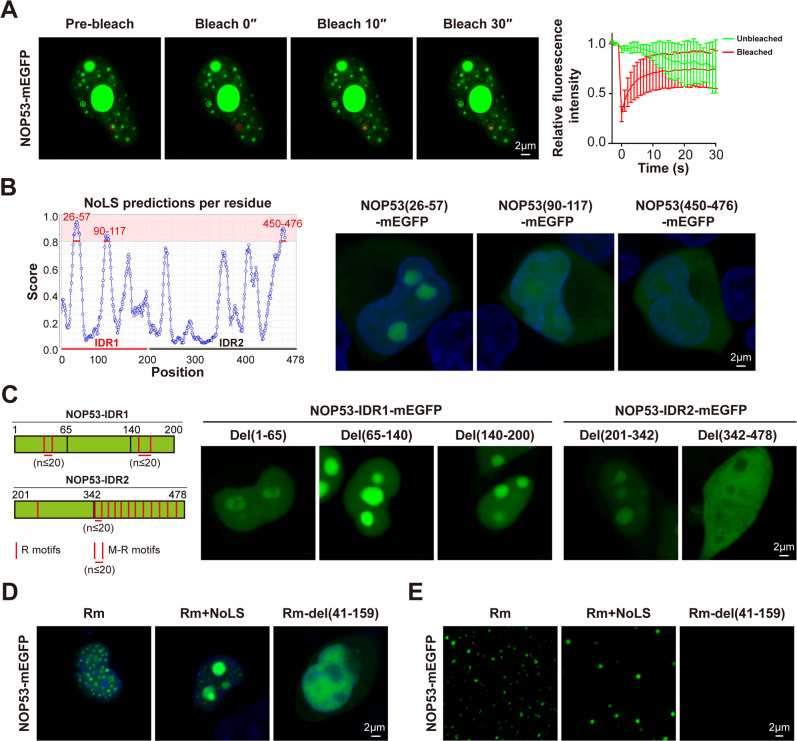
NOP53 undergoes LLPS independent of the nucleolus. **A** FRAP of NOP53-mEGFP puncta in the nucleoplasm of HEK293T cells. **B** The NoLS of NOP53 was analyzed with NOD (www.compbio.dundee.ac.uk/software.html#3Dstructure) and verified in HEK293T cells. **C** Schematic of R motifs or M-R motifs distribution in NOP53 sequence and truncation of NOP53-IDR1/IDR2 sequence. R motifs were defined as the sequence pattern, RX_n1_R (n1 ≤ 2), while M-R motifs were defined as the sequence pattern, RX_n1_RX_n2_RX_n3_R (n1 ≤ 2, n3 ≤ 2, and n2 ≤ 20). **D** Distribution of NOP53-Rm-mEGFP, NOP53-Rm+NoLS-mEGFP and NOP53-Rm-del(41-159)-mEGFP in HEK293T cells. **E** The purified NOP53-Rm-mEGFP and NOP53-Rm+NoLS-mEGFP protein, but not NOP53-Rm-del(41-159)-mEGFP protein, formed droplets in vitro. One micromolar protein was used.

In addition to the large NOP53 puncta referring to the nucleoli, many small puncta containing rapid FRAP were observed in the nucleoplasm of NOP53-mEGFP-overexpressing HEK293T cells (Fig. 4A), indicating that NOP53 may undergo LLPS independent of nucleoli. We next investigated the nucleoli-localizing motif of NOP53. Among all three potential nucleolar localization sequences (NoLS) of NOP53 protein, only the sequence of 26-57 amino acid (a part of IDR1) was characterized as a NoLS (Fig. 4B). Furthermore, M-R motifs, another sequence associated with nucleolar localization [28], were also found in NOP53 (Fig. 4C). R motifs were defined as the sequence pattern, RX_n1_R (n1 ≤ 2), while M-R motifs were defined as the sequence pattern, RX_n1_RX_n2_RX_n3_R (n1 ≤ 2, n3 ≤ 2, and n2 ≤ 20). To investigate whether M-R motifs are necessary for the nucleolar localization of NOP53, a series of truncated mutants of NOP53 were expressed in HEK293T cells. As shown in Fig. 4C, NOP53 mutants containing M-R motifs could localize in the nucleolus even without NoLS. We then investigated whether NOP53 underwent LLPS after eliminating its nucleolar localization by removing NoLS and M-R motifs. Interestingly, although mutation of all R motif and M-R motifs (designated as R motifs mutant, Rm) disrupted the nucleolar localization of both full-length and IDR1 of NOP53, they formed large number of droplets in nucleoplasm (Fig. 4D and Supplementary Fig. 4A). Besides, deleting 41-159 amino acid of NOP53, a part of IDR1, diminished the LLPS of Rm, consistent with our observation that IDR1 was the driver of NOP53 condensation (Fig. 4D and Supplementary Fig. 4A). The results from in vitro studies using purified recombinant protein further confirmed this conclusion (Fig. 4E and Supplementary Fig. 4B). Together, these data demonstrate that NOP53 undergoes LLPS independent of the nucleolar localization.

### Abrogation of NOP53 sensitizes tumor cells to radiation by stimulating p53 pathway

**Fig. 5:**
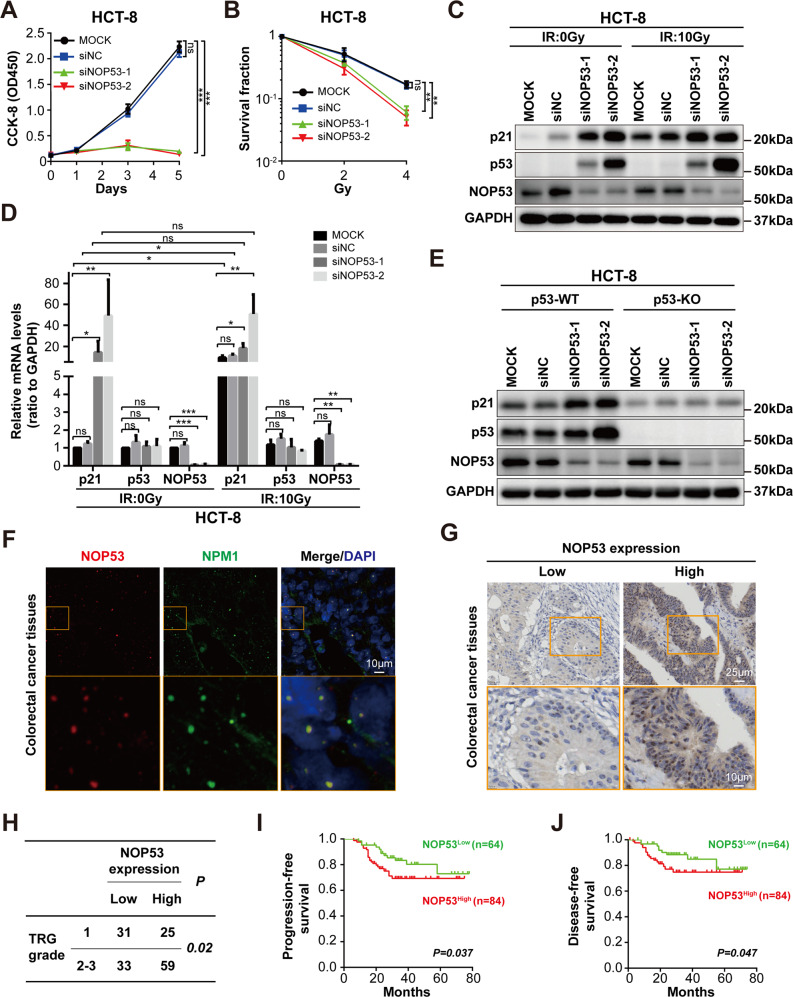
NOP53 promotes tumor radio-resistance. **A** CCK-8 assay showed that NOP53 silencing abolished the growth of HCT-8 cells. **B** Colony formation assay showed that NOP53 enhanced the radio-resistance of HCT-8 cells. **C**, **D** NOP53 silencing increased p53 and p21 protein (**C**) and mRNA (**D**) level in HCT8 cells treating with/without irradiation. HCT-8 cells were transfected with NOP53 siRNA for 48 h before irradiation (10 Gy X-rays), then cultured for 24 h before western blotting analysis. **E** NOP53 depletion-induced p21 upregulation was dependent on p53. **F** NOP53 was localized to the nucleolus in CRC tissues. **G**, **H** CRC patients with higher NOP53 level were more resistant to radio-chemotherapy. TRG, Tumor regression grade. **I**, **J**. Higher NOP53 expression was correlated with a shorter progression-free survival and disease-free survival time of CRC patients. For (**G**–**J**), CRC tissues from 148 patients were analyzed. Data are expressed as mean ± SD; *,*P* < 0.05; **,*P* < 0.01; ***,*P* < 0.001; ns, no significance.

To explore the function of NOP53, NOP53 was silenced via small interfering RNA (siRNA). The silencing efficiency was verified by Western blotting (Supplementary Fig. 5A and Supplemental Material). Cell Counting Kit-8 (CCK-8) assay demonstrated that NOP53 knockdown resulted in significant proliferation inhibition in HCT-8 cells (Fig. 5A). Moreover, NOP53 knockdown sensitized HCT-8 cells to RT in an in vitro colony formation assay (Fig. 5B). Nucleoli play pivotal roles in the stress-induced activation of p53 [29]. Therefore, we hypothesized that NOP53 may enhance radio-resistance by suppressing irradiation-induced p53 activation. In agreement with our hypothesis, silencing of NOP53 largely increased the protein level of p53 and its target gene p21, which was further intensified after irradiation (Fig. 5C, Supplementary Fig. 5B and Supplemental Material). Knockdown of NOP53 had little effect on p53 mRNA level, whereas it significantly increased the mRNA levels of p21 (Fig. 5D). Furthermore, NOP53 silencing had no effect on p21 protein level in p53-depleted HCT-8 cells, suggesting that NOP53 regulated p21 by interfering p53 (Fig. 5E, Supplementary Fig. 5C and Supplemental Material). Together, these results indicate that NOP53 suppresses p53 pathway and enhances radio-resistance of CRC cells.

### NOP53 high expression was associated with the radio-resistance and poor prognosis of CRC patients

We next analyzed the association between NOP53 expression and RT response of CRC patients. Immunofluorescence analysis on CRC frozen sections showed that NOP53 was colocalized with nucleolar protein NPM1, which was consistent with the observation in CRC cell line (Fig. 5F). Next, immunohistochemistry (IHC) assay was performed to analyze NOP53 expression on surgical resection specimens of 148 CRC patients receiving neoadjuvant chemoradiotherapy (Fig. 5G). As shown in Fig. 5H, high expression of NOP53 was correlated with the poor response of neoadjuvant chemoradiotherapy (*P* = 0.02). Also, higher NOP53 level was correlated with shorter progression-free survival (PFS) (Fig. 5I) and disease-free survival (DFS) time (Fig. 5J). Interestingly, radiation enteritis tissues displayed lower expression of NOP53 than those of non-radiation enteritis (*P* = 0.009) (Supplementary Fig. 5D, E), indicating that NOP53 played pivotal roles in radio-sensitivity of both tumoral and normal colorectal tissues.

## Discussion

In this study, we showed that NOP53 underwent liquid-liquid phase separation in the nucleoli, which was mediated by its IDR1. Meanwhile, the M-R motifs within NOP53 mediated its localization in the nucleolus. Moreover, we found that NOP53 inhibited radiation-induced p53 pathway and enhanced tumor radio-resistance. Our data indicate that NOP53 may be an effective therapeutic target for radio-resistant cancer patients.

The nucleolus is a multilayered biomolecular condensate consisting of FC, DFC, and GC [30]. Many nucleolar proteins, including FBL and NPM1, have been highlighted to be phase separated and contribute to the nucleolus assembly [31, 32]. NPM1 is a prominent protein within the GC and known to have a central role in nucleolar structure. It promotes the LLPS of nucleoli through a multi-modal mechanism including multivalent interactions with proteins containing M-R motifs and rRNA [28]. Besides, NPM1 can also undergo LLPS via homotypic interactions between its acidic- and basic-tracts within its IDR [32]. In this study, we showed that NOP53 resided predominantly in the GC and was co-localized with NPM1, which was ascribed to the presence of dozens of M-R motifs in NOP53 protein. By removing all M-R/R motifs, NOP53 was presented only in nucleoplasm, but still formed LLPS condensates, indicating that NOP53 underwent LLPS independent of nucleoli, instead of just being recruited to nucleoli by NPM1.

Whether NOP53 is a suppressor or enhancer of cancer malignancy remains controversial. Previously, researchers reported that NOP53 was involved in the death and transformation of tumor cells [33]. NOP53 bound to and stabilized the tumor suppressor PTEN and induced apoptotic cell death [34]. In addition, NOP53 was a key upstream regulator of p53; it directly stabilized p53 in alternate reading frame (ARF)-deficient cells [24]. Other evidence, however, suggests that NOP53 may promote tumor malignancy. Akira Suzuki et al. showed that NOP53 regulated ribosomal protein–p53 pathway in response to nucleolar stress and that loss of NOP53 inhibits tumor growth owing to stabilization of p53 [25, 35]. Moreover, for human cancer patients with wild-type p53, lower level of NOP53 was associated with a better prognose [25, 36]. Our findings showed that NOP53 promoted CRC cell growth and radio-resistance by suppressing p53 activation in both normal and irradiated cells. Most importantly, the tumorigenic role of NOP53 was further confirmed with clinical samples that CRC patients highly expressing NOP53 had a shorter survival time. However, the exactly role of LLPS in NOP53-mediated radioresistance remains to be further elucidated.

Enhanced DNA damage response signaling, which includes cell cycle checkpoints activation and DNA repair, has been reported to be a key molecular mechanism underlying tumor radio-resistance [3]. Some DDR factors were found to facilitate cell cycle arrest activation to provide sufficient time for radiation-induced DNA repair, thus conferring radio-resistance [37]. For example, Wang et al. revealed that overexpression of the proto-oncogene c-MYC leads to increased expression of CHK1 and CHK2 and subsequent activation of the DNA-damage-checkpoint response, resulting in radio-resistance [38]. Lee et al. demonstrated that as a key DDR sensor, the MRN (MRE11-RAD50-NBS1) complex was found overexpressed in rectal cancer and its high expression was associated with radio-resistance and poor prognosis [39]. Consistently, targeting RAD50 increases the sensitivity to RT in CRC cells [40]. In this study, we showed that NOP53 was overexpression in CRC and associated with radio-resistance and poor prognosis. Specially, we found that NOP53 was largely decreased in radiation enteritis tissues, indicating that NOP53 decreased the radio-sensitivity of both tumoral and normal cells. Therefore, how to specifically target NOP53 in tumor tissues and avoid the influence of normal tissues should be further studied.

Taken together, we identified NOP53 forming liquid-liquid phase separated condensates in the nucleoli. NOP53 is important for cell growth and radio-resistance of CRC cells via negatively regulates p53 activation. Importantly, high level of NOP53 suggested a poor response to RT of CRC patients, indicating NOP53 as a potential target for enhancing tumor radio-sensitivity.

## Materials and methods

### Cell lines and clinical samples

HEK293T, Hela, and HCT-8 cell lines were purchased from American Type Culture Collection (ATCC). U2OS cell line was purchased from Guangzhou Cellcook Biotech Co., Ltd. All cell lines were mycoplasma-free and were authenticated using STR profiling by the provider ATCC or Cellcook. HEK293T, HeLa and U2OS cells were cultured in Dulbecco’s modified Eagle’s medium (DMEM, Gibco, ThermoFisher Scientific, Waltham, Massachusetts, USA), while HCT-8 cells were cultured in RPMI 1640 medium (Gibco). Cells were maintained in culture medium supplemented with 10% fetal bovine serum (FBS, Gibco) at 37 °C under 5% CO_2_. p53 knocked-out (p53-KO) HCT-8 cell line was generated using CRISPR/cas9, and the sequence of small guide RNA (sgRNA) was 5’-TCGACGCTAGGATCTGACTG-3’. mEGFP knocked-in (mEGFP-KI) U2OS cell line was constructed as described by Samie R Jaffrey et al. [41]. The sequence of the gRNA was 5’-AGGTGAAGCTGGTGGAGAAG-3’. We generated a donor vector containing 1070-and 328-nucleotide-long homology arms flanking a mEGFP-P2A-Puro^r^ coding sequence immediately before the stop codon of NOP53. The sequences of primers are given in Supplementary Table 1.

The sequences of siRNAs of NOP53 used in this study are given in Supplementary Table 2. Transfections of siRNA into cells were carried out with Lipofectamine RNAiMAX (56532, Invitrogen) according to the manufacturer’s instructions.

All clinical samples were collected from the Tissue Bank of the Sixth Affiliated Hospital, Sun Yat-sen University. The study was approved by Human Medical Ethics Committee of the Sixth Affiliated Hospital of Sun Yat-sen University and informed consent was obtained from each patient.

### Plasmid constructs

To generate GFP/mEGFP-tagged, mCherry-tagged or Cry2-mCherry-tagged plasmids, NOP53, IDR1, IDR2, RPA49, FBL, or NPM1 fragments were cloned by PCR using human cDNA as template and inserted into pcDNA3.0 vector in frame. pcDNA3.0-NOP53-Rm-mEGFP was constructed commercially (Tsingke, Guanzhou, China). We used the KOD-Plus-Mutagenesis Kit (SMK-101, TOYOBO, Kita-ku, Osaka, Japan) to generate the following mutants: NOP53 fragments containing amino acids 26–57, 90-117, and 450–476; NOP53-IDR1 with a deletion of amino acids 1–65, 65–140, and 140–200; NOP53-IDR2 with a deletion of amino acids 201–342 and 342–478; NOP53-Rm+NoLS-mEGFP, NOP53-Rm-del(41-159)-mEGFP, NOP53-Rm-del(51-159)-mEGFP, NOP53-Rm-IDR1-mEGFP, NOP53-Rm-IDR2-mEGFP, and NOP53-Rm fragments containing amino acids 181–478, 160–478, and 140–478.

The pGEX-NOP53-mEGFP and pGEX-NOP53-Rm-mEGFP expression plasmids were constructed commercially (GENEWIZ, Suzhou, China; Tsingke) for codon optimization. With pGEX-NOP53-mEGFP as a template, NOP53-IDR1 was cloned by PCR into pGEX-GFP vector to generate pGEX-NOP53-IDR1-GFP expression plasmid. Other expression constructs including pGEX-NOP53-Rm+NoLS-mEGFP and pGEX-NOP53-Rm-del(41-159)-mEGFP were created using pGEX-NOP53-Rm-mEGFP as template. To generate p53-KO HCT-8 cell line, a lentiCRISPRv2 vector was used to create a plasmid targeting the *p53* genomic locus. To generate mEGFP-KI U2OS cell line, a pX330 vector was used to create a plasmid targeting the endogenous *NOP53* genomic locus and a pUC19 vector was used to create a donor plasmid containing mEGFP-P2A-Puro^r^ and homology arms. All generated plasmids were sequence-verified.

### Live-cell imaging

All live-cell imaging were carried out on a Zeiss LSM880 confocal microscope equipped with an incubation chamber (37 °C, 5% CO_2_). HEK293T cells were transfected with the plasmid in 35 mm glass-bottom dishes and grown for 36 h. Then, Hoechst 33342 (4082, Cell Signaling Technology, CST, Danvers, MA, USA) was added to the culture medium and the cells were incubated for 10 min at 37 °C before imaging. *ATP depletion*: Cells were cultured in glucose-free DMEM (11966025, Gibco) for 2 h and added with 5 mM 2-deoxy-glucose (HY-13966, MedChemExpress, MCE, Monmouth Junction, NJ, USA) and 126 nM Oligomycin (495455, Sigma-Aldrich, St. Louis, Missouri, USA) for another two hours incubation before observation. *1,6-hexanediol treatment*: Cells were grown in culture medium and imaged every 1 s and then replaced with culture medium containing 10% 1,6-hexanediol. After imaging 60 s, the culture medium was replaced with complete medium for additional image acquisition. *Blue light-inducible droplets formation*: Cells transfected with pcDNA3.0-NOP53-Cry2–mCherry, pcDNA3.0-NOP53-IDR1-Cry2-mCherry or pcDNA3.0-NOP53-IDR2-Cry2-mCherry plasmid after 24 h were recorded time-lapse imaging with light pulses at 488 nm (blue light, 50% laser power) every 2 s/0.6 s/1 s. *Subcellular localizations of NOP53*: Cells were transfected with pcDNA3.0-NOP53-mEGFP plasmid together with RPA49-mCherry, FBL-mCherry or NPM1-mCherry plasmid, respectively, and incubated at 37 °C for 36 h before imaging.

### Immunofluorescence

For cellular immunofluorescence, cells were seeded on 24 wells plate with the slides for 24 h and thereafter fixed with 4% paraformaldehyde (DF0135, Leagene, Beijing, China) for 15 min at room temperature. The coverslips were then treated with blocking buffer (1×PBS containing 5% goat serum and 0.3% Triton X-100) for 1 h and incubated with primary antibodies overnight at 4 °C. After three washes with PBS, cells were incubated with Alexa fluor-conjugated 488 or 555 secondary antibodies (4408S, 4413S, CST) for 1 h at room temperature in the dark, followed by three washes in PBS and staining with DAPI for 5 min (D9542, Sigma-Aldrich). Glass slides were mounted in ProLong™ Diamond Antifade Mountant (P36965, Invitrogen). For frozen section immunofluorescence, the sections were fixed with acetone for 10 min at 4 °C, penetrated by 1×PBS containing 0.3% Triton X-100 for 30 min and blocked in blocking buffer (5% goat serum in 1× PBS) for 30 min at 37 °C. Antibodies incubation were then performed as described above. Primary antibodies utilized for immunofluorescence are as follows: anti-NOP53 (73225S, CST, 1:100) and anti-NPM1 (60096-1-Ig, Proteintech, Rosemont, IL, USA, 1:500).

### Fluorescence recovery after photobleaching (FRAP)

FRAP was performed using LSM-880 confocal microscope (Zeiss) with the 488 nm laser. Bleaching was performed at 100% laser power, and images were collected every 1 second. The entire puncta or part of the puncta inside was photobleached during time-lapse imaging. Images were further processed, and the fluorescence intensity in the photobleached region was measured using ZEN3.1 (Blue Edition) and values were normalized to pre-bleach time points.

### Protein expression and purification

pGEX-NOP53-mEGFP plasmid and other NOP53 fragments plasmids were transformed into *E. coli* strain BL21 (DE3) cells, respectively. Cultures were grown at 37 °C until the OD600 reached 0.6–0.8 and then induced by adding 0.5 mM isopropyl beta‐d‐thiogalactopyranoside (IPTG) for growth at 16 °C overnight. The next day, cells were pelleted by centrifugation at 4000 × *g* for 10 min at 4 °C followed by resuspending in lysis buffer (20 mM Tris-HCl, pH 7.5, 150 mM NaCl, 10% glycerol and 1 mM dithiothreitol (DTT)) and adding 1 mM phenylmethanesulfonyl fluoride (PMSF) before cells lysing by sonication (power setting of 50%, 120 × 5 s with 5 s intervals). After centrifugation at 10,000 × *g*, 4 °C for 10 min, the supernatant was incubated with GST-tagged purification resin (SA008100, Smart-Lifesciences, Changzhou, China) at 4 °C for 2 h. Then, resin was washed well with GST lysis buffer and NOP53 protein was eluted with glutathione (GSH) elution buffer [20 mM Tris-HCl, pH 7.5, 150 mM NaCl, 10% glycerol, 1 mM DTT and 25 mM GSH]. The eluted protein was digested with human rhinovirus type 14 3 C protease (P2303, Beyotime) and purified by HiTrap Heparin HP/Capto HiRes Q/Superdex 200 Increase columns (17040701/29275878/28990944, Cytiva, Marlborough, MA). Finally, proteins were frozen in high salt buffer (50 mM Tris-HCl, pH = 7.5; 1 M NaCl) and stored at −80 °C.

### In vitro droplet assay

Recombinant NOP53-mEGFP, NOP53-IDR1-GFP, and GFP proteins were first adjusted to varying temperatures with indicated concentration in buffers containing 20 mM Tris-HCl (pH = 7.4) and 150 mM NaCl. At the appropriate temperature tested above, proteins were diluted to varying concentrations in buffers containing 20 mM Tris-HCl (pH = 7.4) and 150 mM NaCl. Then different of protein concentrations, salt concentrations and pH were performed as changed conditions for further experiments. All the protein solutions (20 µL) were incubated at indicated temperature for 10 min in PCR tubes and loaded onto glass slides. Slides were then imaged on Zeiss LSM880 confocal microscope with a 64x oil objective and further processed by ZEN software (Blue edition, 3.1). Fluorescence intensity was measured by Image J.

### Cell counting Kit-8 assay

HCT-8 cells were divided into four groups. Each group was seeded on 96-well plates with 2 × 10^3^ cells per well and transfected with siNC, siNOP53-1, and siNOP53-2, respectively. The rest of the group served as a blank control. Cultivation was performed at 37 °C. The CCK-8 solution (10 μL) was added to each well at the indicated time points (0, 1, 3, and 5 days) and incubated for 3 h at 37 °C. Then quantified the absorbance at 450 nm.

### Colony formation assay

HeLa cells were divided into four groups for experiment. Each group was seeded on a six-well plate with 3000 cells/well and transfected with siNC, siNOP53-1, and siNOP53-2, respectively. The rest of the group served as a blank control. The next day, each group was irradiated with various doses of 0 Gy, 2 Gy, and 4 Gy. Colonies were stained with crystal violet and counted using Image J 11 days after irradiation.

### Western blot analysis

HCT-8 cells were lysed in RIPA buffer (25 mM Tris-HCl, pH = 7.4; 150 mM NaCl; 1% NP-40; 0.5% Na-deoxycholate) supplemented with protease inhibitor cocktail. Proteins were separated by 10–12% SDS–PAGE, blotted onto PVDF membrane and incubated with primary antibodies (mouse anti‐GAPDH, 60004-1-Ig, Proteintech, 1:5000; rabbit anti-NOP53, CST, 1:1000; rabbit anti-p53, 10442-1-AP, Proteintech, 1:1000; rabbit anti-p21, 10355-1-AP, Proteintech, 1:1000) overnight at 4 °C, followed by incubation with appropriate secondary antibody (Goat Anti-Rabbit IgG(H + L) HRP, GAR0072, MULTISCIENCES and Goat Anti-Mouse IgG(H + L) HRP, GAM0072, MULTISCIENCES, Hangzhou, China, 1:5000) for 1 h at RT. The protein intensity was analyzed using Image Lab v5.2.1.

### Real-time quantitative polymerase chain reaction (qPCR)

According to manufacturer’s instructions, total RNA was extracted using TRIzol reagent (15596018, Invitrogen). For reverse transcription we used ReverTra Ace® qPCR RT Master Mix with gDNA Remover (FSQ-301, TOYOBO). All the qPCR were performed on a LightCycler 480 System (Roche, Basel, Switzerland) using SYBR® Green Realtime PCR Master Mix (QPK-201, TOYOBO). Between duplicate wells, cycle threshold (Ct) values differed by less than 0.5. Normalizing the relative expression levels of the target genes to those of internal control genes, we obtained a 2^-ΔCt^ value. GAPDH was used as a gene for reference. The sequences of primers are given in Supplementary Table 3.

### Immunohistochemistry and analysis

Tissue samples used in this study were derived from CRC patients who received postoperative neoadjuvant chemoradiotherapy and radiation enteritis patients. Formalin-fixed paraffin-embedded samples were sliced into 5 μm sections and mounted on polylysine-coated slides. After incubation in an oven at 55 °C until paraffin melted, tissue slides were deparaffinized in xylene followed by rehydration in graded alcohol. Slides were soaked in citrate buffer (10 μM, pH = 6.0, ZSGB-BIO, Beijing, China) and heated in a microwave processor for antigen retrieval in radiation enteritis tissues. For CRC tissues, slides were immersed in EDTA buffer (pH = 9.0, ZSGB-BIO) and antigens were retrieved using hyperbaric heating. After naturally cooling to room temperature (RT), tissue samples were incubated in hydrogen peroxide (0.3%) in the dark for 10 min to block endogenous peroxidase activity. Slides were then incubated with anti-NOP53 antibody (73225S, CST, 1:200) overnight at 4 °C in a humidified chamber, followed by incubating with Biotin-Streptavidin HRP Detection System (SP-9000, ZSGB-BIO) for 35 min at 37 °C. Then slides were stained with a DAB Detection Kit (ZLI-9018, ZSGB-BIO) for 5 min at room temperature before they were stained with haematoxylin (Zymed Laboratories, South San Francisco, CA, USA), and subsequently dehydrated, mounted, and covered with coverslips. Images were acquired using a slide scan system. Samples were classified according to the percentage of positive cells: 0 (0%), 1 (<25%), 2 (26–50%), 3 (51–75%) and 4 (>76%); and staining intensity in: 0, negative; 1, weak signal; 2, moderate signal; and 3, strong signal. Addition of scores estimated from the positive percentage and intensity of staining, the final score was calculated for each sample: score 1 (a final score of 0–1); score 2 (2–3); score 3 (4–5); and score 4 (a final score of 6–7), which was then categorized as low expression (final score was 1 and 2) and high expression (final score was 3 and 4).

### Statistical analysis

Statistical analysis were conducted using SPSS 20.0 software (SPSS Inc., Chicago, IL, USA). Data were represented as mean ± standard deviation of independent experiments performed in triplicate. Differences between two groups were assessed by unpaired *t* test. The Kaplan–Meier method and the log-rank test were used to analyze overall survival. For the study of association between NOP53 expression and TRG grade as well as that between NOP53 expression and the presence or absence of radiation enteritis, chi-square test was performed. *P* < 0.05 were considered significant.

## Supplementary information

Supplementary Figure and Legend

Supplementary Table

Original Data File

## Data Availability

All data generated or analyzed during this study are included in this published article (and its supplementary information files). Source data are provided with this paper.
