# Supplementary material for: NOP53 undergoes liquid-liquid phase separation and promotes tumor radio-resistance
Source: Cell Death Discov. 2022 Oct 31;8:436. doi: 10.1038/s41420-022-01226-8 (PMC9622906; doi:10.1038/s41420-022-01226-8)
Supplement: Supplementary file 1 — Supplementary Figure and Legend [file 41420_2022_1226_MOESM1_ESM.docx]

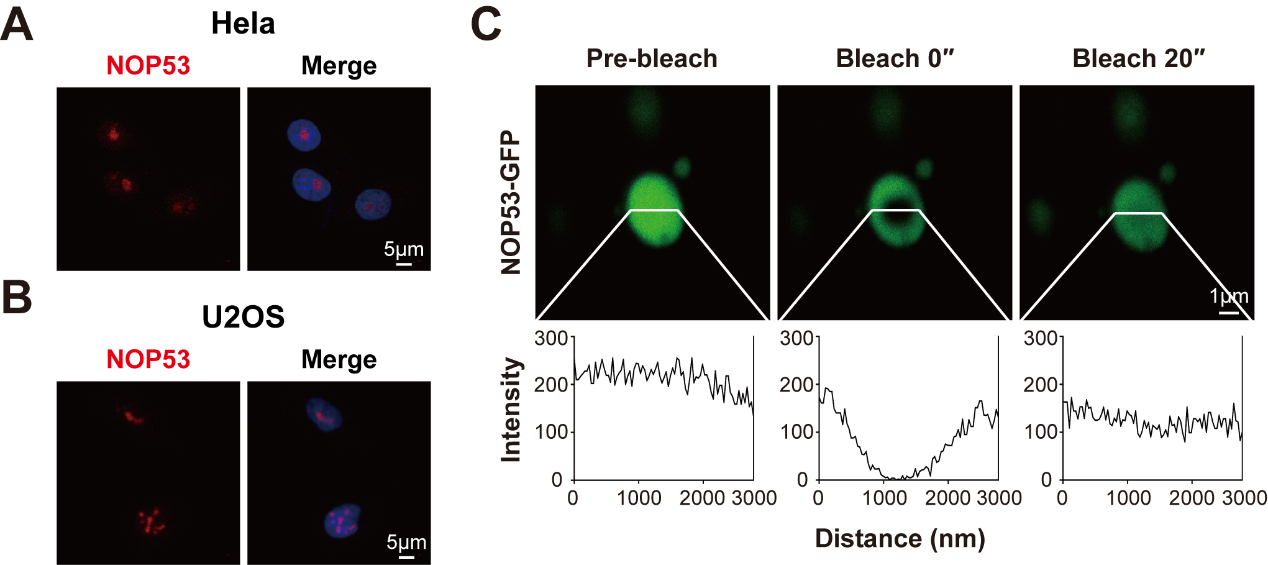


**Supplementary Figure 1. NOP53 was localized in nucleoli and showed liquid-liquid phase separation property in cells. Related to Figure 1.** A. Immunofluorescence of endogenous NOP53 in HeLa cells. B. Immunofluorescence of endogenous NOP53 in U2OS cells. C. FRAP of a region within the NOP53 droplets in live HEK293T cells.


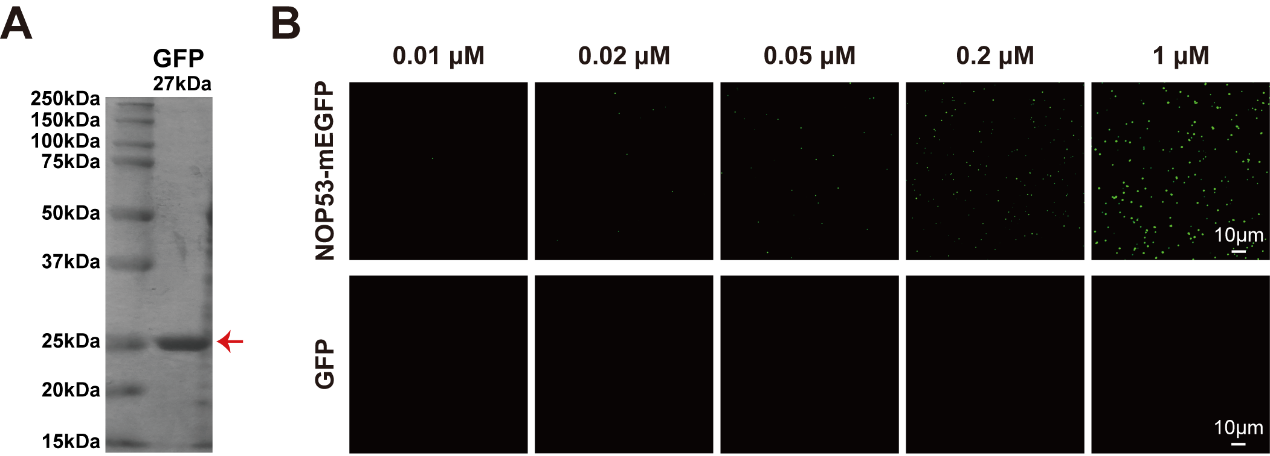


**Supplementary Figure 2. Recombinant NOP53-mEGFP undergoes LLPS *in vitro*. Related to Figure 2.** A. Coomassie staining of purified GFP protein. B. NOP53-mEGFP droplets that formed in buffers containing 150 mM NaCl and 25 mM Tris-HCl (pH7.4) were observed with confocal microscopy.


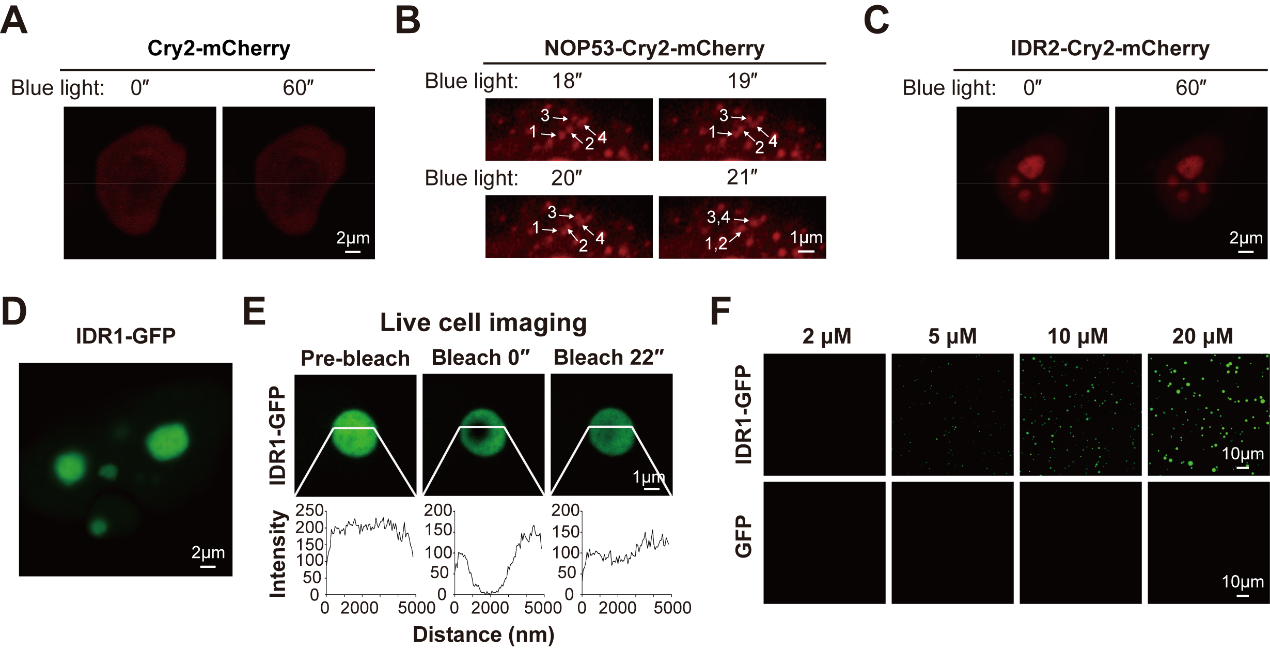


**Supplementary Figure 3. IDR1 drives the LLPS of NOP53. Related to Figure 3.** A. Cry2-mCherry was expressed in cells and stimulated with blue light. B. NOP53-Cry2-mCherry recombinant protein formed droplets upon blue light stimulation, and the fusion of adjacent droplets was observed in cells. C. IDR2-Cry2-mCherry was expressed in cells and stimulated with blue light to induce condensation. D. IDR1-GFP showed puncta in the nucleolus of HEK293T cells. E. FRAP of a region within the NOP53-IDR1 droplets in live HEK293T cells. **F**. The impact of protein concentration on the formation of NOP53-IDR1-GFP droplets.


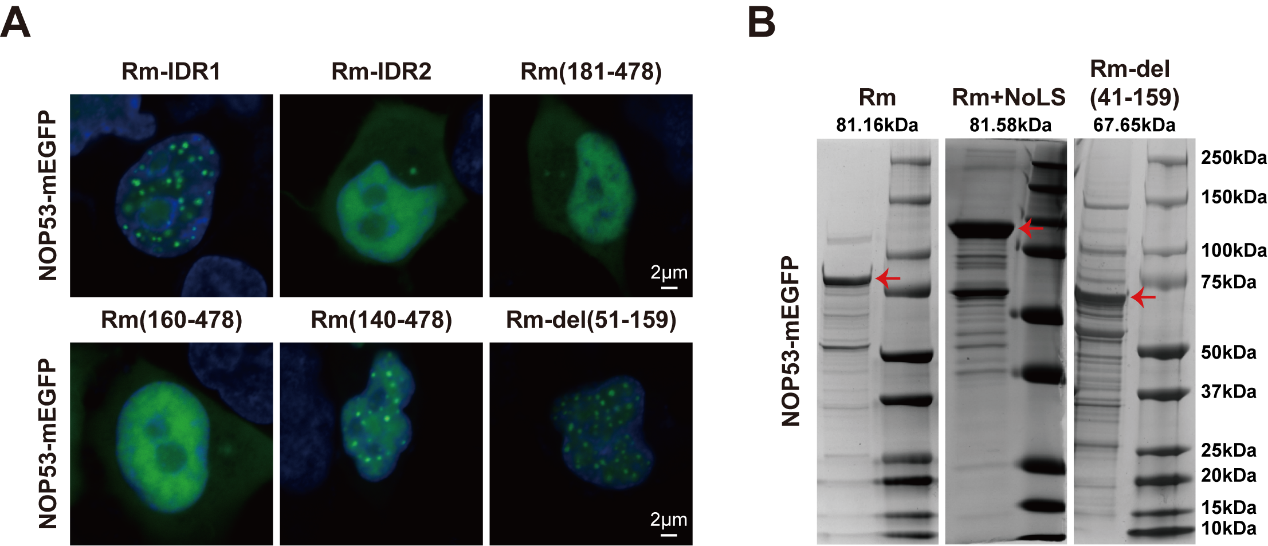


**Supplementary Figure 4. NOP53 undergoes LLPS independent of the nucleolus. Related to Figure 4.** A. Expression of different NOP53 mutants in HEK293T cells. B. Coomassie staining of purified NOP53-Rm-mEGFP, NOP53-Rm+NoLS-mEGFP and NOP53-Rm-del(41-159)-mEGFP protein.


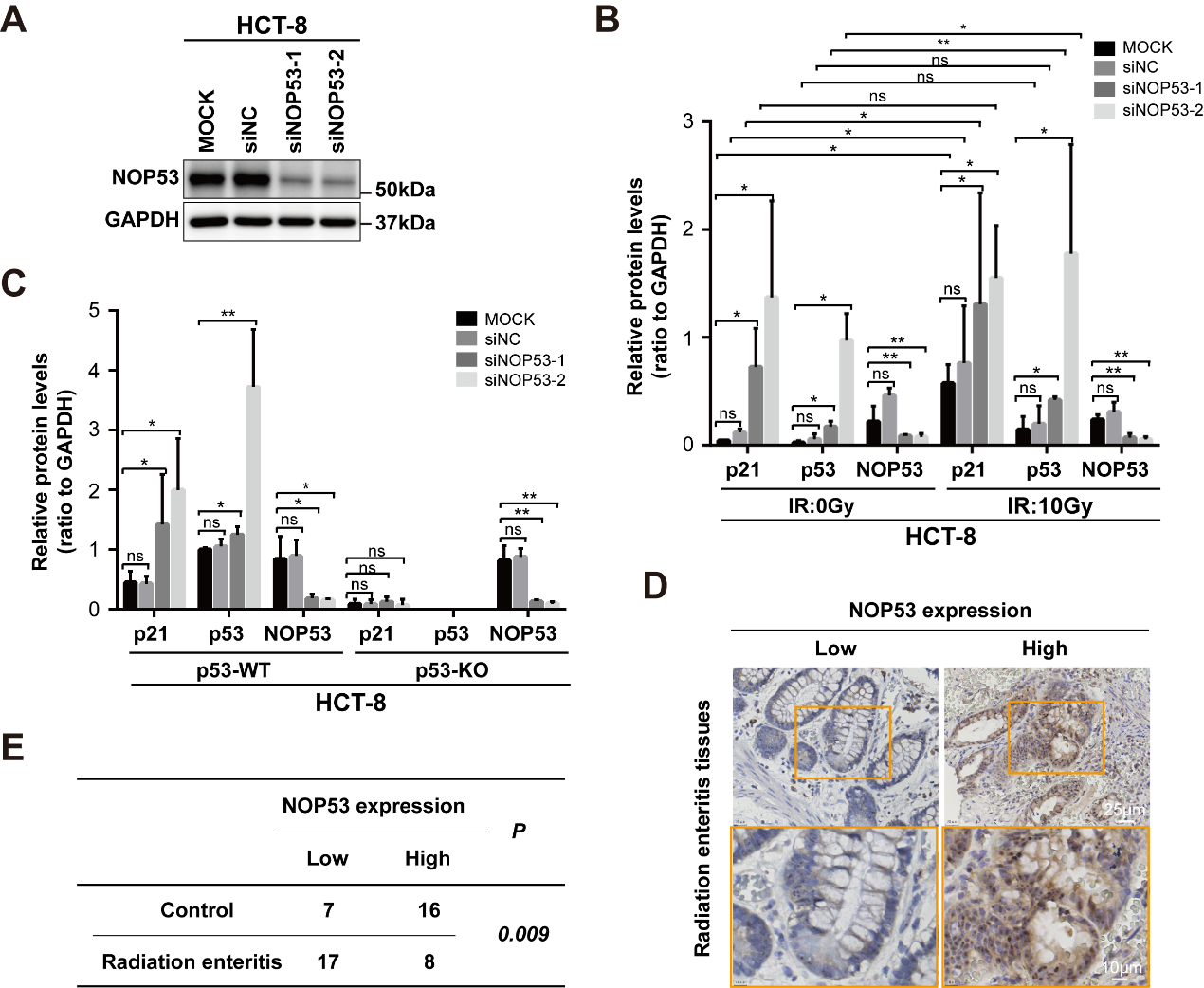


**Supplementary Figure 5. NOP53 promotes tumor radio-resistance. Related to Figure 5.** A. HCT-8 cells were transfected with siRNA to specifically knock down NOP53 and the expression of NOP53 was determined by Western blotting. B-C. Relative intensity analysis of Western blotting image in Figure 5C and 5E, respectively. D-E. NOP53 was downregulated in radiation-induced intestinal injury tissues. Data are expressed as mean ± SD; *, *P* < 0.05; **, *P* < 0.01; ns, no significance.
