# Supplementary material for: NOP53 undergoes liquid-liquid phase separation and promotes tumor radio-resistance
Source: Cell Death Discov. 2022 Oct 31;8:436. doi: 10.1038/s41420-022-01226-8 (PMC9622906; doi:10.1038/s41420-022-01226-8)
Supplement: Supplementary file 2 — Supplementary Table [file 41420_2022_1226_MOESM2_ESM.docx]

**Supplementary Table 1. Sequences of primers for donor plasmid**

| Primers | 5’-3’ |
| --- | --- |
| 5’HR-F  5’HR-R  mEGFP-F  Puro-R  3’HR-F  3’HR-R | GATCTACTAGTCATATGGATTGGGGCCAATGCCCAGGGG  CAACCTGGGGACAGAGGAAAG  TTTCCTCTGTCCCCAGGTTGGTGAGCAAGGGCGAGGAGC  TCACGGAACGCCCGCTTTCAGGCACCGGGCTTGCG  TGAAAGCGGGCGTTCCGTGAG  CTCGGTACCCGGGGATCCGATCTCCCAACTCCGGCCAGG |

**Supplementary Table 2. Sequences of siRNAs**

| siRNA |  | 5’-3’ |
| --- | --- | --- |
| NOP53-1 | sense | GGGCUGACAAAGAAGAGAATT |
|  | antisense | UUCUCUUCUUUGUCAGCCCTT |
| NOP53-2 | sense | GGAGCUUCCUACAAUCCAUTT |
|  | antisense | AUGGAUUGUAGGAAGCUCCTT |
| NC | sense | UCACAACCUCCUAGAAAGAGUAGA |
|  | antisense | UACUCUUUCUAGGAGGUUGUUAUU |

**Supplementary Table 3. Sequences of primers for qPCR**

| Primers | 5’-3’ |
| --- | --- |
| NOP53-F  NOP53-R  p21-F  p21-R  p53-F  p53-R  GAPDH-F  GAPDH-R | GTACCAGGCACCTGACATCG  ACCTTGTACTTGCGTTTGAACT  GACCATGTGGACCTGTCACT  GATTAGGGCTTCCTCTTGGA  GGAAGGAAATTTGCGTGTGG  CCAGTGTGATGATGGTGAGG  GAGTCAACGGATTTGGTCGT  GACAAGCTTCCCGTTCTCAG |
