# Supplementary material for: NOP53 undergoes liquid-liquid phase separation and promotes tumor radio-resistance
Source: Cell Death Discov. 2022 Oct 31;8:436. doi: 10.1038/s41420-022-01226-8 (PMC9622906; doi:10.1038/s41420-022-01226-8)

Original image: Figure 1E

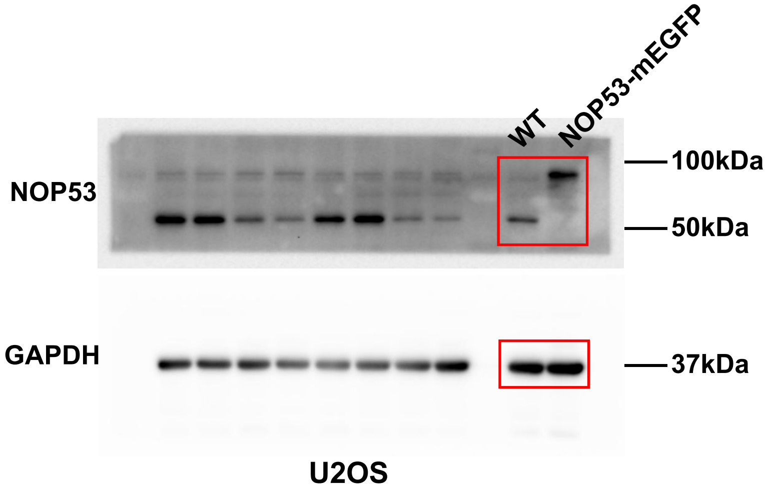

Original image: Supplementary Figure 5A

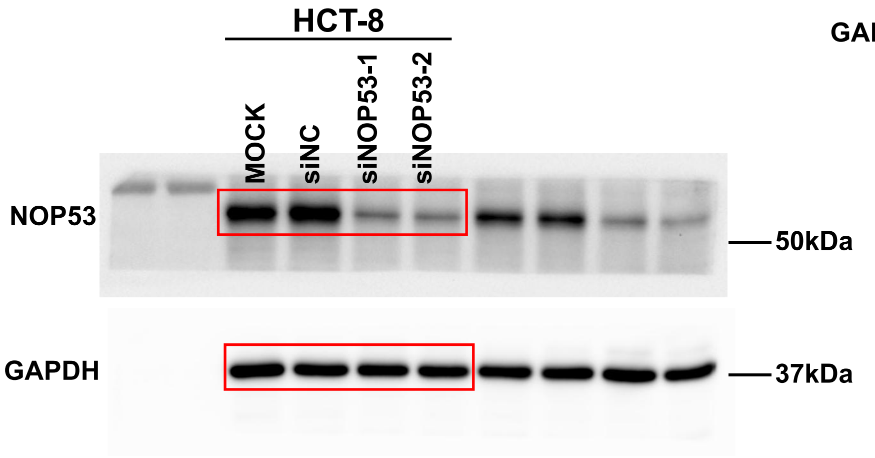

Original image: Figure 5C

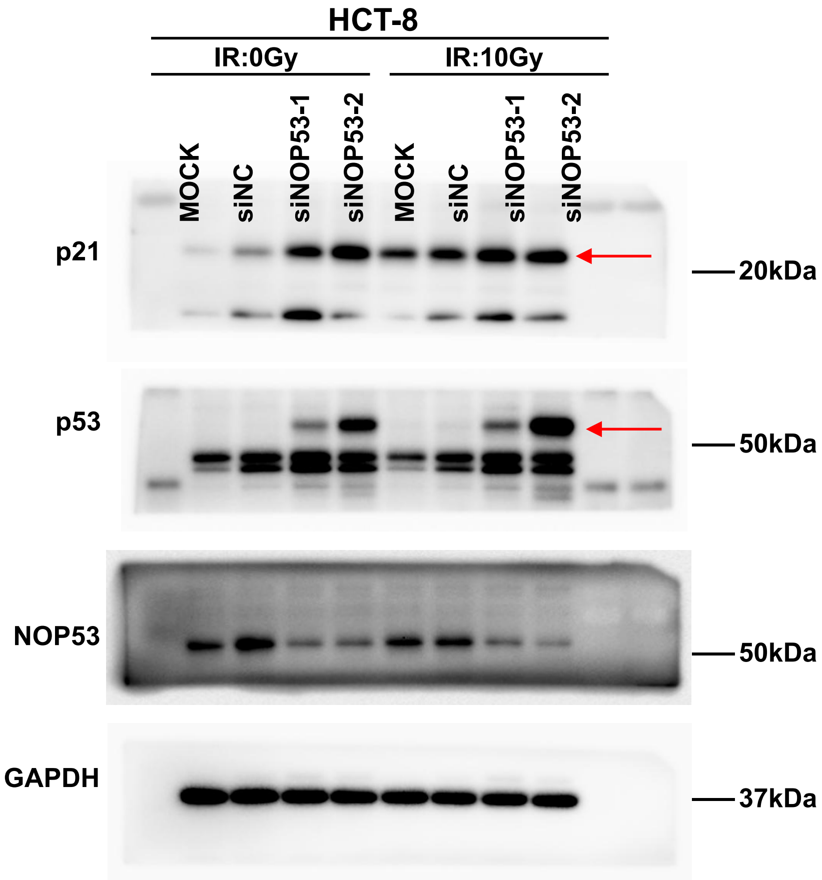

Original image: Figure 5E

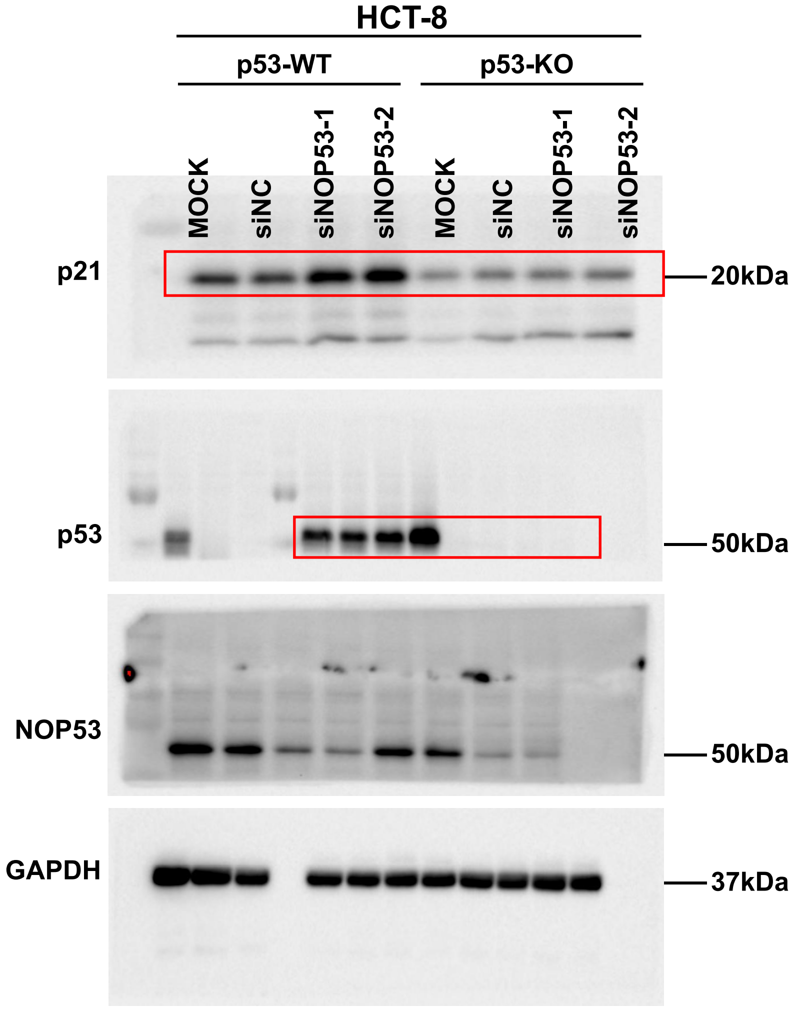

Supplement: Supplementary file 3 — Original Data File [file 41420_2022_1226_MOESM3_ESM.pdf]
